# Time to Differentiate Postactivation “Potentiation” from “Performance Enhancement” in the Strength and Conditioning Community

**DOI:** 10.1007/s40279-020-01300-0

**Published:** 2020-06-03

**Authors:** Olaf Prieske, Martin Behrens, Helmi Chaabene, Urs Granacher, Nicola A. Maffiuletti

**Affiliations:** 1 University of Applied Sciences for Sports and Management Potsdam, Am Luftschiffhafen 1, 14471 Potsdam, Germany; 2 Institute of Sport Science, University of Rostock, Rostock, Germany; 3 Division of Training and Movement Sciences, Research Focus Cognitive Sciences, University of Potsdam, Potsdam, Germany; 4 Human Performance Lab, Schulthess Clinic, Zurich, Switzerland

## Abstract

Coaches and athletes in elite sports are constantly seeking to use innovative and advanced training strategies to efficiently improve strength/power performance in already highly-trained individuals. In this regard, high-intensity conditioning contractions have become a popular means to induce acute improvements primarily in muscle contractile properties, which are supposed to translate to subsequent power performances. This performance-enhancing physiological mechanism has previously been called postactivation potentiation (PAP). However, in contrast to the traditional mechanistic understanding of PAP that is based on electrically-evoked twitch properties, an increasing number of studies used the term PAP while referring to acute performance enhancements, even if physiological measures of PAP were not directly assessed. In this current opinion article, we compare the two main approaches (i.e., mechanistic vs. performance) used in the literature to describe PAP effects. We additionally discuss potential misconceptions in the general use of the term PAP. Studies showed that mechanistic and performance-related PAP approaches have different characteristics in terms of the applied research field (basic vs. applied), effective conditioning contractions (e.g., stimulated vs. voluntary), verification (lab-based vs. field tests), effects (twitch peak force vs. maximal voluntary strength), occurrence (consistent vs. inconsistent), and time course (largest effect immediately after vs. ~ 7 min after the conditioning contraction). Moreover, cross-sectional studies revealed inconsistent and trivial-to-large-sized associations between selected measures of mechanistic (e.g., twitch peak force) vs. performance-related PAP approaches (e.g., jump height). In an attempt to avoid misconceptions related to the two different PAP approaches, we propose to use two different terms. Postactivation potentiation should only be used to indicate the increase in muscular force/torque production during an electrically-evoked twitch. In contrast, postactivation performance enhancement (PAPE) should be used to refer to the enhancement of measures of maximal strength, power, and speed following conditioning contractions. The implementation of this terminology would help to better differentiate between mechanistic and performance-related PAP approaches. This is important from a physiological point of view, but also when it comes to aggregating findings from PAP studies, e.g., in the form of meta-analyses, and translating these findings to the field of strength and conditioning.

## Key Points

**Table:** 

A mechanistic (e.g., twitch peak force) and a performance-related understanding (e.g., jump height) of PAP have been established in the literature with different characteristics, e.g., in terms of effective conditioning contractions, testing procedures, or time courses of effects.
Associations between selected measures of the mechanistic vs. the performance-related PAP approaches revealed inconsistent trivial-to-large-sized correlation coefficients.
We propose alternative terminology to unambiguously differentiate between increases in muscular force/torque production during an electrically-evoked twitch (postactivation potentiation [PAP]) and enhancements of measures of maximal strength, power, and speed (postactivation performance enhancement [PAPE]) following conditioning contractions.

## Introduction

In elite sport, small performance differences can decide whether an athlete makes it to the podium or not. During the 100 m final at the 2009 Athletics World Championship in Berlin, for example, Usain Bolt achieved an average velocity of 12.3 m s^−1^ over the fastest 60–80-m split distance, contributing to the fabulous world record time of 9.58 s [1]. Interestingly, the average sprinting velocities of the second (Tyson Gay) and third (Asafa Powell) finishers over the same split distance were 12.1 and 11.9 m s^−1^, respectively, which is only 1.6% and 3.3% slower than Usain Bolt’s record. Therefore, coaches and athletes are seeking to use advanced training strategies to further improve physical performance (i.e., components of physical fitness, sport-specific performance) and, thereby, attenuating the apparently small gaps. This is of particular interest with regards to the law of diminishing returns [2], because performances are reduced in high-level compared with novice athletes [2–4]. This is supported by findings from Rhea et al. [4] who showed consistently smaller effect sizes of strength training-related performance gains in trained compared with untrained individuals.

An advanced training strategy for improving powerful performance (e.g., speed), particularly in young and high-level athletes, is to combine maximal or near-maximal strengthening exercises immediately followed by plyometric or ballistic exercises. In the scientific literature, this methodology is usually referred to as complex training [5–8]. There is evidence that complex training revealed the largest beneficial effects on sport-specific performance compared with other types of resistance training (e.g., plyometric training, machine-based resistance training) in young adolescent athletes [8]. Adaptive processes following complex training have been primarily attributed to the long-term translation of acute improvements in muscle contractile properties, induced by preceding high-load strengthening exercises. This performance-enhancing physiological phenomenon is well-known under the term postactivation potentiation (PAP) [6, 7]. However, even though the number of scientific publications on PAP effects is constantly growing, there appears to be a misconception amongst researchers on the proper meaning and usage of PAP. While some researchers use it in the traditional mechanistic understanding that is based on electrically-evoked twitch properties of muscles (e.g., twitch peak force), others extend the notion and refer to performance measures (e.g., vertical jump height, sprint time). For the assessment of twitch contractile properties, highly standardized laboratory-based tests are required [9], while physical performance can easily be quantified using field and lab-based tests [10]. Due to the large heterogeneity of methods that are used to examine PAP effects, data interpretation can be flawed. These inconsistencies could undermine the accuracy of scientific knowledge and, consequently, the professional translation of study findings to the field of strength and conditioning. Therefore, the purpose of this opinion paper was to provide a brief literature review on the main methodological approaches (i.e., mechanistic vs. performance) to study PAP. We additionally aimed at extracting misconceptions in the use of the term PAP by analyzing the relationship between the two PAP approaches. From this, we want to propose that the two main approaches should not be used interchangeably. Finally, alternative definitions will be suggested for future studies and evidence-based exercise programs to unambiguously differentiate the two PAP approaches.

## Background and Putative Approaches to Examine Postactivation Potentiation

**Fig. 1:**
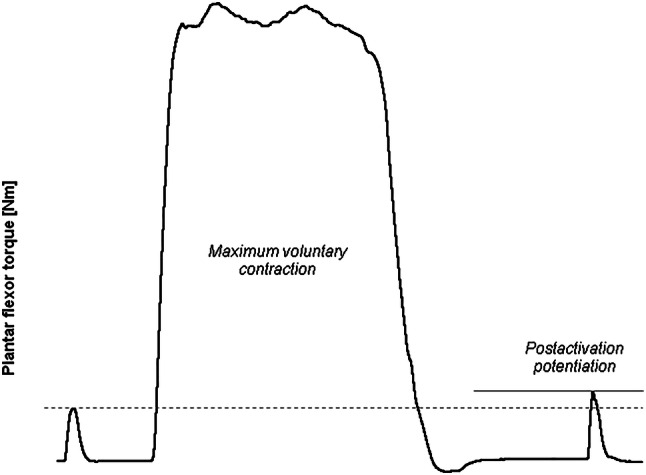
Example of the measurement of postactivation potentiation (PAP) in the plantar flexor muscles. A baseline twitch is artificially evoked in the resting plantar flexors. Two seconds following a conditioning maximum voluntary contraction, the evoked twitch has a greater peak torque compared with the baseline twitch. The increment from baseline twitch peak torque (dashed line) to post-contraction twitch peak torque (solid line) corresponds to the extent of PAP

The PAP phenomenon and its underlying physiological mechanisms have already been studied for many decades. In fact, animal studies from the mid of the nineteeth century showed that tetanic stimulation of the frog’s gastrocnemius muscle significantly enhanced subsequent isometric twitch force measures induced by a single electrical stimulus (for reviews see [11, 12]). Similarly, a series of repeated electrical stimuli revealed progressive increments of adductor pollicis muscle twitch peak force (i.e., staircase) in healthy humans [13]. Moreover, isometric twitch force of the dorsiflexor and plantar flexor muscles was significantly increased immediately after maximal voluntary contractions (MVC) in healthy males and females [14]. In light of the available evidence from studies with animals and human beings, Sale [15] defined PAP as an increase in isometric twitch peak force or low-frequency tetanic force/torque after (1) a series of evoked twitches, (2) an evoked tetanic contraction, or (3) a MVC (i.e., conditioning contraction). According to this definition, the evaluation of PAP effects focuses on muscle contractile properties and requires an electrically-evoked response to ensure that the enhancements occur for the same level of electrical stimulation ([9, 12]; Table 1). Figure 1 shows a typical example of PAP that was induced by a MVC. From a physiological point of view, the main contributor to PAP has been proposed to be the phosphorylation of myosin regulatory light chains [12, 16, 17]. In fact, phosphorylation of regulatory light chains via the myosin light chain kinase increases the sensitivity of the actin–myosin complex to myoplasmic Ca^2+^ resulting in enhanced myosin cross-bridge activity and, therefore, in elevated contractile force/torque production [15, 16]. Other factors such as recruitment of higher order motor units and changes in muscle pennation angle have been discussed more controversially suggesting that the contribution of these mechanisms to PAP is only minor [15–17]. Furthermore, the occurrence of enhanced twitch contractile properties following conditioning contractions has consistently been reported in the literature [9, 16, 18–26]. Nevertheless, the magnitude of twitch force/torque potentiation appears to be influenced by different factors such as the type (e.g., MVC, maximal hopping, submaximal leg press) and volume (i.e., total duration) of the conditioning contraction, rest time after the conditioning contraction, subjects’ characteristics (e.g., sex, age, training status, muscle strength level, fiber-type distribution), muscle length and the number of applied electrical stimuli (i.e., single vs. paired stimuli) [9, 16, 19–23]. In fact, the extent of potentiation for contractile properties ranged from 4 to 188% immediately after the conditioning contraction, with a progressive decline over time ([14, 19, 21, 24–26]; Table 1).

**Table 1:** Comparison between the two approaches of postactivation potentiation

	Mechanistic approach	Performance approach
Research field	Basic research	Applied research, strength and conditioning practice
Conditioning contraction	Isometric, dynamic	Isometric, dynamic
	Stimulated/voluntary	Voluntary
	Single-/multi-joint	Single-/multi-joint
	High-intensity	High-intensity
Verification	Electrical stimulation of single muscles/muscle groups (lab-based tests)	Voluntary contractions during single-/multi-joint exercises (lab-based/field tests)
Effects	↑ Peak twitch force/torque	↑ Maximal strength
	↑ Rate of twitch force/torque development	↑ Jump performance
		↑ Sprint performance
		↑ Performance during explosive actions
Extent	4–188% of pre-CC peak twitch force/torque	1–13% of pre-CC performance
Occurrence	Consistent (all subjects, muscles, and conditions)	Inconsistent
Time course	Exponential decline over ~ 10 min (largest effect immediately after CC)	Initial decline followed by “Gaussian” profile (largest effect ~ 7 min after CC)
Suggested term	Postactivation potentiation (PAP)	Postactivation performance enhancement (PAPE)

*CC* conditioning contraction

Specifications based in the relevant literature ([9, 14–16, 18, 19, 21, 24–26, 36, 53, 56])

**Fig. 2:**
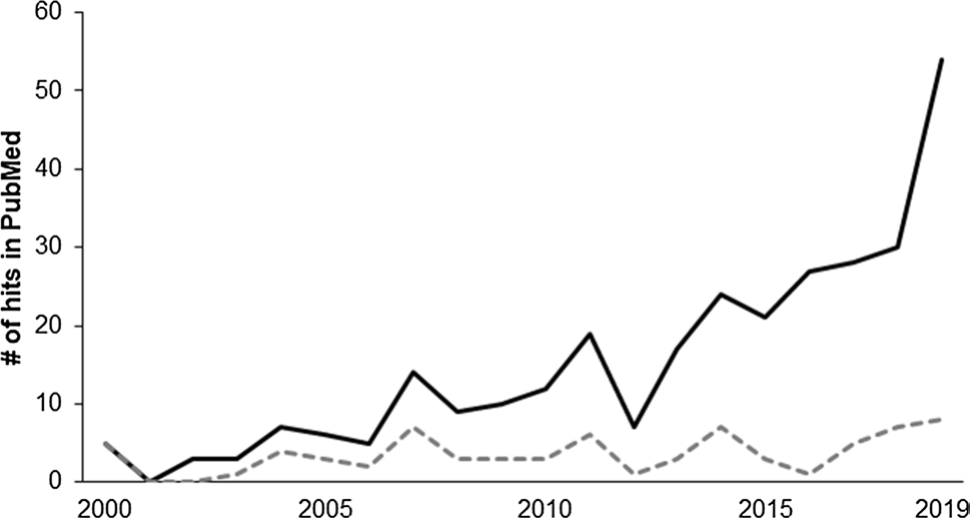
Number of hits on the topic of postactivation potentiation using the online database PubMed. Lines indicate the hits across time for different search strategies on postactivation potentiation alone (*black solid line*: “postactivation potentiation” OR “post-activation potentiation”) or with “twitch” as an additional search term (*grey dashed line*: (“postactivation potentiation” OR “post-activation potentiation”) AND twitch)

The interest in examining PAP effects has increased since the narrative reviews of Sale at the beginning of the Millenium ([15, 27]; see Fig. 2). In this regard, it has to be noted though that the number of studies dealing with PAP effects and evoked twitches as the mechanistic verification remained constant (Fig. 2). In contrast, the scientific understanding of PAP progressively drifted away from the originally mechanistic definition to a more performance-oriented approach [9, 15]. In a constantly increasing number of cross-sectional studies but also in systematic reviews, acute performance enhancements induced by previous conditioning contractions were attributed to PAP effects, even though no direct physiological/mechanistic measures were assessed [28–35]. Performance improvements ranged from 1 to 13% for measures of jumping or sprinting ([36]; Table 1). For instance, McLaren et al. [37] examined the acute effects of 3 sets of loaded back squats at 70% of the 1-repetition maximum (1RM) followed by a rest period of 8 min on a subsequent series of 40-m sprints in male field-sport athletes. They found significant performance gains in up to 3 sprints following the conditioning contraction and concluded that “the PAP effect was sustainable up to 11 min after heavy back squats” [37]. Furthermore, a systematic review with meta-analysis stated that conditioning contractions with multiple sets of strengthening exercises at moderate intensities (60–85% 1RM) and with rest periods of 7–10 min should be used to elicit PAP effects in the form of improved measures of muscle power (e.g., jumping, Wingate test) [35]. Likewise, a recent systematic review with meta-analysis postulated that dynamic movements at ≥ 80% 1RM and rest periods of 3–7 min should be used to enhance PAP effects on vertical jump performance [34]. Interestingly, before peaking at ~ 7 min following the conditioning contractions, performance is initially decreased most likely due to the negative net effect of fatigue and twitch force/torque potentiation and/or movement pattern interference between conditioning contractions and subsequent exercise [12, 15, 18]. These studies consistently showed that, in contrast to the originally suggested definition by Sale [15], the mechanistic factors were marginalized and PAP effects were merely discussed on a performance level.

## What is the Problem with the Misconception of Postactivation Potentiation?

The above-discussed inconsistency in the definition and understanding of PAP (mechanistic vs. performance-related approach) bears fundamental risks for the misinterpretation of study findings. Consequently, basic scientific knowledge and dissemination of study findings to practitioners in the field of strength and conditioning can be distorted. Indeed, some studies assert that the potentiation of twitch contractile properties (e.g., twitch peak torque [TPT]) induced by submaximal and maximal contractions may partly contribute to acute performance enhancements (e.g., increased jump height) [38–41]. For instance, the studies of Mitchell and Sale [38] and Fukutani et al. [41] reported concomitant PAP-related increases in knee extensor TPT (28–40%) and countermovement jump height (3–11%) which occurred 0.5 to 4 min following submaximal squat exercises in trained male adults. The corresponding effect sizes (ES) were medium-to-large (0.54 ≤ ES ≤ 1.37) and small-to-large (0.22 ≤ ES ≤ 0.87), respectively. Additionally, there is evidence that repetitive hopping induced significant and large-sized gains in plantar flexor TPT (1.47 ≤ ES ≤ 3.26) and drop jump height (1.36 ≤ ES ≤ 6.75) 30 s following the conditioning activity in recreationally active individuals [26, 42]. Thus, the authors concluded that twitch PAP effects contributed to gains in jump performance [26, 38, 41, 42]. However, statistical associations between pre-to-post-exercise changes of twitch contractile properties (i.e., mechanistic PAP approach) with strength, power, or speed measures (i.e., performance PAP approach) are inconsistent in the literature. In fact, a number of studies reported trivial-to-large-sized correlation coefficients (|*r*| ≤ 0.61) between changes in TPT of plantar flexors/knee extensors and jump height/kinetics in young female athletes [24] and recreationally-trained individuals [38–40, 42, 43]. These relatively poor and inconsistent associations between changes in twitch contractile properties and the corresponding strength, power, or speed performance indicate that individuals with greater twitch PAP effects in single muscle groups are not necessarily those showing the greatest single-/multi-joint performance improvements following acute exercise. In fact, other studies observed PAP effects following high-intensity contractions (e.g., MVC, submaximal leg press) but no acute performance changes [24, 44–46]. In female young elite soccer players, submaximal exercises on a leg press resulted in large-sized enhancements in twitch rate of torque development (ES = 1.98) 7 min following conditioning contractions compared with a passive control condition [24]. However, no significant improvements were found in countermovement and drop jump performances. Notably, a sequence of double-leg balance and submaximal leg press exercises induced significantly higher countermovement jump heights and shorter drop jump ground contact times (1.82 ≤ ES ≤ 1.98) 7 min following conditioning contractions compared with a passive control condition [24]. However, no significant differences were observed in twitch contractile properties. Interestingly, it was suggested that PAP can only effectively contribute to performance enhancements within 1–5 min after conditioning contractions [47]. Having these findings and the temporal decline in the effects of PAP in mind, it seems legitimate to state that factors other than twitch PAP may predominantly contribute to the acute performance enhancements. These factors could most likely be related to general warm-up effects which are observed a few minutes following conditioning contractions. In this context, various physiological effects, e.g., warm-up related changes in muscle temperature, metabolism, baseline oxygen consumption, muscle activation, motor learning, and even subjects’ psychological state were reported to induce acute and transient enhancements in physical performance (for reviews see [12, 18]). For instance, higher muscle temperatures due to exercise can reduce the viscous resistance of muscles and joints and increase nerve conduction velocity [18]. Elevated baseline oxygen consumption following warm-up (e.g., conditioning contractions) may allow individuals to reduce anaerobic demands during the first stages of the subsequent tasks [18]. Furthermore, exercise may acutely potentiate selected neuromuscular responses (e.g., H-reflex) [24, 48]. Moreover, MacIntosh and colleagues [47] discussed the learning effect as a major confounding factor in studies dealing with PAP effects. More precisely, performance could be acutely enhanced by learning how to do the performance test, particularly with unfamiliar tests/tasks [12, 47]. Additionally, studies on motor learning showed that practicing one task (e.g., conditioning contractions) can transfer to another, similar task (i.e., skill transfer) [49, 50]. In this regard, beneficial effects of repetitive hopping, for instance, on subsequent drop jumps may also be attributed to skill transfer due to similar motor patterns (i.e., stretch–shortening cycle; [51]). Thus, it is highly speculative and potentially misleading to attribute acute performance enhancements following conditioning contractions exclusively to twitch PAP effects.

Furthermore, it should be noted that twitch PAP following conditioning contractions may also be overestimated for acute performance improvements, because each muscle action during the targeted exercises can induce PAP (and fatigue) effects itself. For instance, Hamada et al. [52] used a fatigue protocol of repetitive isometric knee extensor MVCs and revealed a progressively increasing potentiation of knee extensor TPT during the first three MVC trials in young males. In another study, knee extensor TPT increased during 3 and 4 sets of dynamic squats with larger increments following 4 compared with 3 sets in Olympic weightlifters [41]. These findings indicate that single muscle actions (isometric or dynamic) during the targeted exercises can induce and even accumulate PAP effects themselves. Therefore, the role of PAP effects of preceding conditioning contractions for subsequent performance enhancements can be questioned.

## How to Solve the Problem with the Postactivation Potentiation Terminology?

The important question that has to be faced and answered is how can researchers and practitioners in the field of strength and conditioning prevent the misconceptions between PAP effects and performance enhancements in the future? In an effort to solve this problem and in accordance with a recent narrative review [12], we suggest to consistently use the terms “postactivation potentiation” (PAP, when referring to the mechanistic approach) vs. “postactivation performance enhancement” (PAPE, when referring to the performance approach). In this regard, PAP has previously been defined as the increase in electrically-evoked twitch force/torque (e.g., higher TPT) following submaximal and maximal conditioning contractions [15]. In contrast, PAPE was suggested to indicate the enhancement of maximal voluntary (dynamic or isometric) strength, power, or speed following a conditioning contraction [53]. These enhancements of maximal and powerful performances are typically represented by improved strength or jumping and sprinting exercises [35, 54]. The term “potentiation” should not be used in the context of acute performance enhancements. When adhering to these definitions (also see Table 1), future studies could define and specify their PAP approach (mechanistic vs. performance) more clearly and discuss their findings more adequately with regard to the applied approach. Moreover, differentiating between PAP and PAPE is particularly important when it comes to aggregating and translating findings from PAP studies to the field of strength and conditioning. For instance, the inconsistency of the mechanistic and performance-related PAP approaches can affect internal validity and, thereby, increase the risk of bias in meta-analyses [55]. Consequently, inferences for practitioners could be misleading.

## Conclusions

Basic research on the potentiation of electrically-evoked (twitch) contractile properties of skeletal muscles following muscular activity in addition to applied research on the effects of exercise on subsequent performance measures do not support the colloquial meaning of the term PAP. Researchers, as well as practitioners in the field of strength and conditioning, should avoid using the term PAP arbitrarily unless the specific definitions and the respective methodologies are taken into consideration. The term PAP can be used to indicate the increase in muscular force/torque production during an electrically-evoked twitch (e.g., higher TPT), whereas PAPE can be used to refer to enhancements in maximal strength, power, and speed following conditioning contractions. With respect to the translation of study findings to strength and conditioning programs, we encourage the future use of this terminology to better differentiate the two PAP approaches and to precisely determine the relationship between mechanistic and performance measures following acute exercise.
